# The inspection paradox: An important consideration in the evaluation of rotor lifetimes in cardiac fibrillation

**DOI:** 10.3389/fphys.2022.920788

**Published:** 2022-09-06

**Authors:** Evan V. Jenkins, Dhani Dharmaprani, Madeline Schopp, Jing Xian Quah, Kathryn Tiver, Lewis Mitchell, Feng Xiong, Martin Aguilar, Kenneth Pope, Fadi G. Akar, Caroline H. Roney, Steven A. Niederer, Stanley Nattel, Martyn P. Nash, Richard H. Clayton, Anand N. Ganesan

**Affiliations:** ^1^ College of Medicine and Public Health, Flinders University, Adelaide, SA, Australia; ^2^ College of Science and Engineering, Flinders University, Adelaide, SA, Australia; ^3^ Department of Cardiovascular Medicine, Flinders Medical Centre, Adelaide, SA, Australia; ^4^ School of Mathematical Sciences, University of Adelaide, Adelaide, SA, Australia; ^5^ Montréal Heart Institute and Université de Montréal, Montréal, QC, Canada; ^6^ School of Medicine, Yale University, New Haven, CT, United States; ^7^ School of Engineering and Materials Science, Queen Mary University of London, London, United Kingdom; ^8^ School of Biomedical Engineering and Imaging Sciences, Kings College London, London, United Kingdom; ^9^ Auckland Bioengineering Institute, University of Auckland, Auckland, New Zealand; ^10^ Insigneo Institute for in Silico Medicine and Department of Computer Science, University of Sheffield, Sheffield, United Kingdom

**Keywords:** ventricular fibrillation, atrial fibrilation, renewal theory, inspection paradox, cardiac fibrillation, phase singularity

## Abstract

**Background and Objective:** Renewal theory is a statistical approach to model the formation and destruction of phase singularities (PS), which occur at the pivots of spiral waves. A common issue arising during observation of renewal processes is an inspection paradox, due to oversampling of longer events. The objective of this study was to characterise the effect of a potential inspection paradox on the perception of PS lifetimes in cardiac fibrillation.

**Methods:** A multisystem, multi-modality study was performed, examining computational simulations (Aliev-Panfilov (APV) model, Courtmanche-Nattel model), experimentally acquired optical mapping Atrial and Ventricular Fibrillation (AF/VF) data, and clinically acquired human AF and VF. Distributions of all PS lifetimes across full epochs of AF, VF, or computational simulations, were compared with distributions formed from lifetimes of PS existing at 10,000 simulated commencement timepoints.

**Results:** In all systems, an inspection paradox led towards oversampling of PS with longer lifetimes. In APV computational simulations there was a mean PS lifetime shift of +84.9% (95% CI, ± 0.3%) (*p* < 0.001 for observed vs overall), in Courtmanche-Nattel simulations of AF +692.9% (95% CI, ±57.7%) (*p* < 0.001), in optically mapped rat AF +374.6% (95% CI, ± 88.5%) (*p* = 0.052), in human AF mapped with basket catheters +129.2% (95% CI, ±4.1%) (*p* < 0.05), human AF-HD grid catheters 150.8% (95% CI, ± 9.0%) (*p* < 0.001), in optically mapped rat VF +171.3% (95% CI, ±15.6%) (*p* < 0.001), in human epicardial VF 153.5% (95% CI, ±15.7%) (*p* < 0.001).

**Conclusion:** Visual inspection of phase movies has the potential to systematically oversample longer lasting PS, due to an inspection paradox. An inspection paradox is minimised by consideration of the overall distribution of PS lifetimes.

## Introduction

The repetitive creation and annihilation of spiral vortices is a pattern observed in spatiotemporally turbulent nonequilibrium systems throughout nature (Lechleiter et al., 1991; Cross and Hohenberg, 1993; Ouyang and Flesselles, 1996; Cross and Greenside, 2009; Tan et al., 2020). In physical (Morris et al., 1993; Ouyang and Flesselles, 1996; Egolf et al., 2000), chemical (Beta et al., 2006; Qiao et al., 2009), and biological systems (Lechleiter et al., 1991; Tan et al., 2020), this motif, variously known as defect-mediated turbulence (Coullet et al., 1989; Ouyang and Flesselles, 1996) or spiral defect chaos (Morris et al., 1993; Egolf et al., 2000), is characterised by the repetitive creation and annihilation of topological defects called phase singularities (PS), which are located at the pivot of spiral waves (Winfree, 1980; Winfree, 1987).

A surprising finding is that the population dynamics of PS in these diverse systems appear to be governed by common statistical laws (Davidsen et al., 2008; Tan et al., 2020). Specifically, theoretical studies have predicted that due to the effective statistically independent nature of spiral nucleation and annihilation processes under conditions of spatiotemporal turbulence, PS populations should follow Poisson distributions (Gil et al., 1990; Wang, 2004), with the corollary being that PS lifetimes should be exponentially distributed (Tan et al., 2020; Quah et al., 2021). These statistical properties have empirically been subsequently demonstrated to apply to spiral vortex regeneration in fluid mechanics (Ecke and Hu, 1997; Egolf et al., 2000), chemical reactions (Beta et al., 2006), quantum fluids (Abo-Shaeer et al., 2002; Tan et al., 2020), as well as biologically in the brain (Huang et al., 2010) and cell membranes of living cells (Tan et al., 2020).

There are strong reasons to suggest that a similar process of regeneration of spiral vortices sustains fibrillation in the heart (Christoph et al., 2018). Unstable re-entrant circuits, are at present considered most likely to arise from spiral vortices, (Comtois et al., 2005) which has been observed in cardiac fibrillation for over a century (Comtois et al., 2005). Exponential distributions of PS lifetimes are observed in pre-clinical mapping data in both atrial (Chen et al., 2000a; Kuklik et al., 2016; Child et al., 2018) and ventricular fibrillation (Rogers et al., 1999; Chen et al., 2000b; Rogers, 2004; Kay and Rogers, 2006; Christoph et al., 2018) (AF/VF), computer simulations of AF and VF and in humans mapped with extracellular electrograms and optical mapping (Chen et al., 2000a; Chen et al., 2000b; Rogers, 2004; Kuklik et al., 2016; Child et al., 2018; Christoph et al., 2018; Schlemmer et al., 2018). Importantly, this principle has been shown to apply to both short-lasting PS and more sustained PS associated with rotors (Rogers et al., 1999; Kay et al., 2006; Dharmaprani et al., 2019b). Collectively, these results suggest, the statistical properties of PS in cardiac fibrillation are similar to those observed to other forms of defect-mediated turbulence in nature.

We have recently investigated spiral vortex regeneration population dynamics in AF and VF (Dharmaprani et al., 2019b; Dharmaprani et al., 2021; Dharmaprani et al., 2022). These studies have shown in cardiac fibrillation, similar to other natural systems, PS inter-formation times and lifetimes fit with an exponential distribution, consistent with the notion that PS creation and annihilation may be characterised as renewal processes (Dharmaprani et al., 2019b; Dharmaprani et al., 2021; Quah et al., 2021). By combining the renewal rate constants for PS formation (λf—pronounced ‘lambda-f’) and destruction (λd), we have shown that it is possible to explain the Poisson distribution of PS that is observed in both AF and VF (Dharmaprani et al., 2019b; Dharmaprani et al., 2021; Quah et al., 2021) (Jenkins et al., 2022).

The finding that PS formation and destruction may be characterised as renewal processes has important implications in the experimental evaluation of cardiac fibrillation. In this study, we consider the potential for an inspection paradox. The inspection paradox is a common issue observed with renewal processes (Angus, 1997; Ross, 2014). Inspection paradoxes occur in scenarios when a renewal process is randomly observed in time, giving rise for the tendency to observe an interval larger than that of an average interval (Ross, 2014). To be specific about what this would mean in AF or VF, this would give the tendency for recordings to commence during longer periods of re-entry, giving the potential to perceive that observed PS lifetimes are longer than the actual average PS lifetime (Angus, 1997; Ross, 2014). The inspection paradox could have the potential to influence the interpretation of mechanistic and clinical studies of cardiac fibrillation, by inadvertently emphasising the relative temporal significance of more sustained re-entrant circuits during the early stages of cardiac fibrillation recordings. This may be relevant, by contributing to the cognitive perception of temporal stability of re-entry.

In the following study, we sought to quantify the potential effect of an inspection paradox on the perception of PS lifetimes through sampling of the lifetimes at possible commencement points for fibrillation recordings. This involved comparing the statistical properties of PS lifetimes sampled across full epochs, with PS lifetimes present during potential commencement timepoints, and was performed across a wide range of experimental and clinical datasets, including computational simulations, animal, and human AF and VF.

## Materials and methods

The potential influence of an inspection paradox is illustrated and examined in two parts. In Part 1–Theory, we demonstrate the effects of an inspection paradox on a simulated renewal process, to demonstrate to readers the operation of an inspection paradox. In Part 2, we simulate the effect of observations around randomly chosen commencement timepoints on PS lifetime distributions in a range of cardiac fibrillation data.

### Part 1–theory

To illustrate how an inspection paradox works, we present data from a simple simulated renewal process generated as follows. Using MATLAB, two exponential probability distributions were generated, each defined by a rate constant, $\lambda_{f}$ / $\lambda_{d}$ . Random draws from the probability distribution defined by $\lambda_{f}$ were used to simulate inter-formation timings of theoretical events (theoretically identical to PS). Random draws from the distribution defined by $\lambda_{d}\;$ were used to define event destruction timings. These timings were used to create a data table of event formation and destruction times, as a continuous sequence of events forming and being destroyed.

**FIGURE 1:**
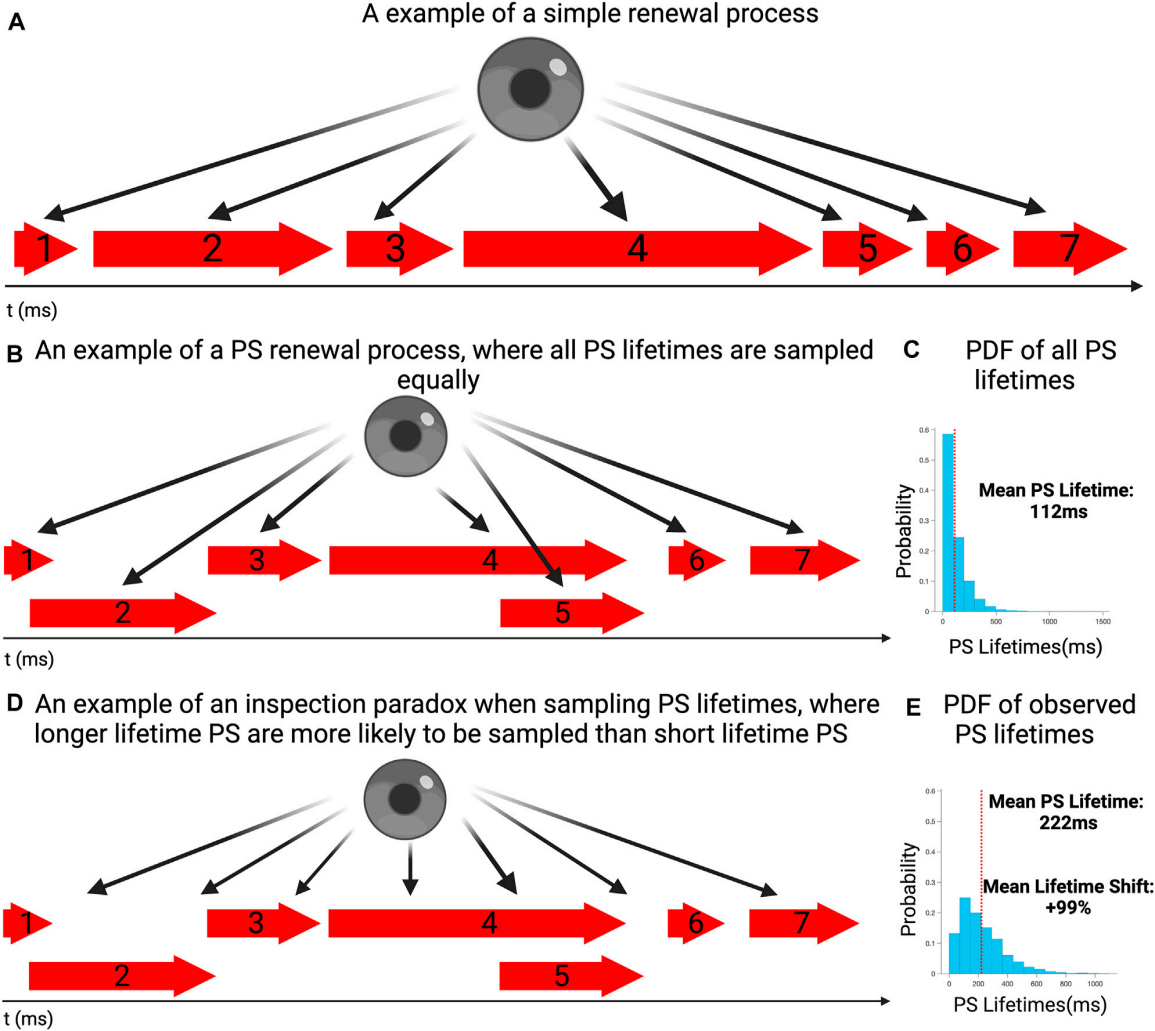
**(A)** An example of a simple renewal process in which event lasts for a period of time, before a new occurs. **(B)** An example of a PS renewal process, where at any given time either one event could be ongoing, multiple events can occur simultaneously or no events could be active. Each event is sampled equally and individually. **(C)** The probability distribution of event lifetimes for a simulated sequence of event lifetimes (PS), consistent with other renewal processes and our previous work. The mean PS lifetime is 112 ms. **(D)** An example where PS lifetimes are sampled according to the moment of observation, rather than properly examining the full distribution of lifetimes around it. Due to an inspection paradox, it is more likely for longer lasting events to be observed rather than short lasting ones. **(E)** To demonstrate how an inspection paradox can lead to a bias in favor of longer lasting events, 10,000 randomly chosen commencement timepoints where chosen, with the lifetimes of PS occurring at those timepoints sampled. The probability distribution demonstrates suggests that longer lasting events had a higher probability of occurring, compared with the overall distribution This is reflected in the mean lifetime of this distribution being 222 ms.

To demonstrate the effect of observation of events around a commencement point, a random time-point in the data-table between 0% and 90% of the final event formation time was selected. The lifetimes of events existing at the randomly chosen timepoint in the data selected were examined, Figures 1A,C.

To demonstrate the potential for a distorting influence, this was repeated many times to create a data set of sampled lifetimes, of events existing at the commencement timepoint. This was performed 10,000 times, to ensure that the distribution of sampled (observed) lifetimes could have a distinct shape. The justification for a 10,000-fold repetition was that a large sampling would ensure characterisation of the shape of the potential distribution developed by the inspection paradox.

In this example, the full distribution of lifetimes of all events is presented in Figure 1B, showing the exponential distribution of lifetimes, with a mean event lifetime of 112 ms. This distributional shape was in keeping with those we have previously identified with renewal processes (Dharmaprani et al., 2019b; Dharmaprani et al., 2021; Dharmaprani et al., 2022; Jenkins et al., 2022). Figure 1D presents the distribution of observed lifetimes, all sampled events, including potential repetitions of events. It can be seen that the mean lifetime in the observed distribution is shifted to the right, with a mean of 222 ms (+99% shift). This demonstrates the disproportionate likelihood of observing longer lifetime events, relative to the true distribution of lifetimes, when examined across a full epoch.

The MATLAB code used to perform this example is provided in the Supplementary Material S1.

### Part 2–the effect of an inspection paradox in atrial and ventricular fibrillation

To examine the impact of an inspection paradox on the observed lifetimes of PS, we examined PS lifetimes. A multi-system, multi-modality study was performed in order to examine the potential influence of an inspection paradox in cardiac, independent of the mapping modality utilized. We first examined it in computational models (a model of spiral-defect turbulence; Aliev-Panfilov (APV) model, followed by more detailed models of AF in 2D and 3D) and then subsequently evaluated them in a range of experimentally and clinically acquired AF and VF data.

**FIGURE 2:**
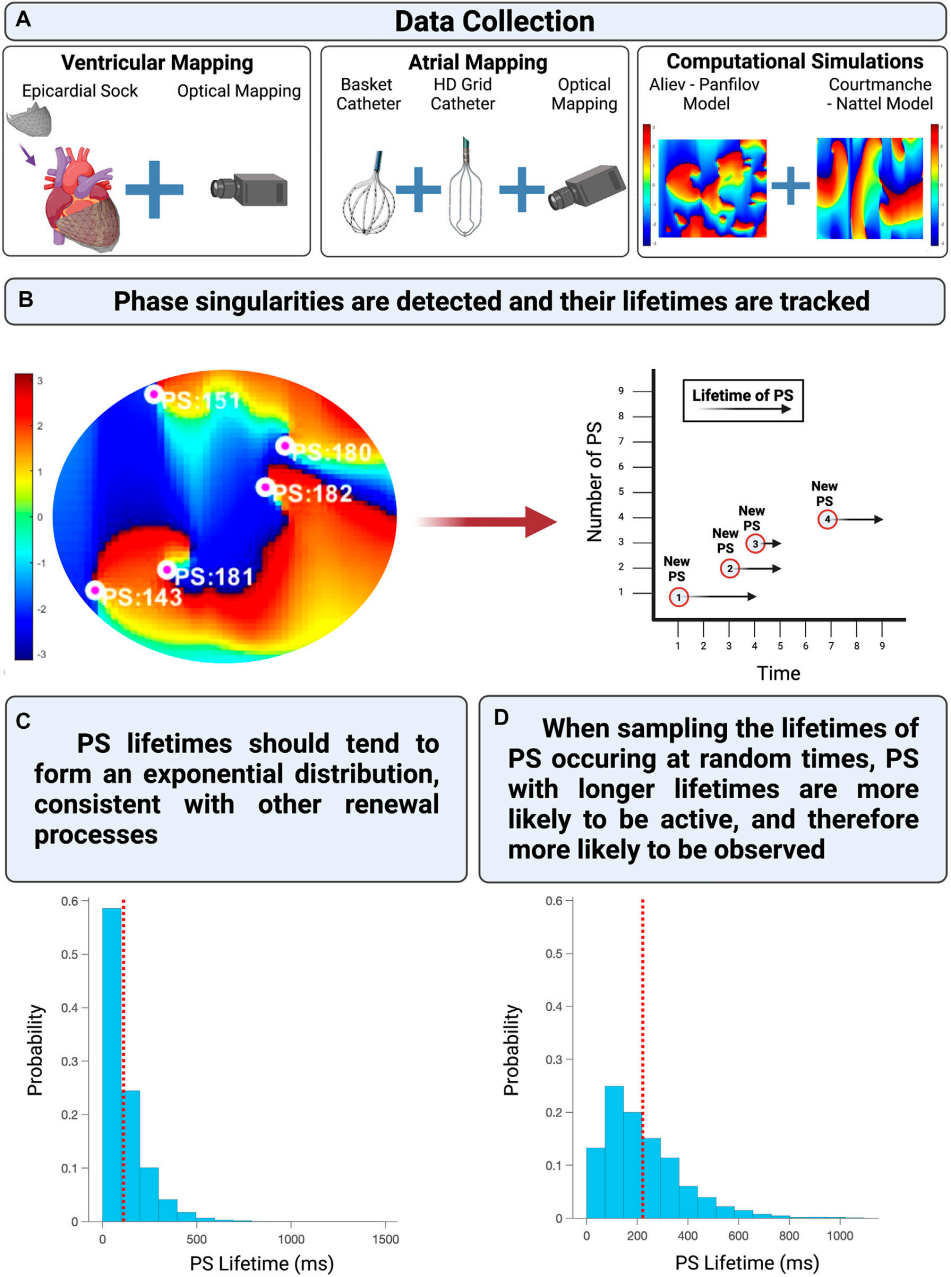
**(A)** The experimentally acquired data, clinically acquired data, and computational simulations utilized in this study. **(B)** Shows presents an example of PS detected and tracked on a phase map. **(C)** Demonstrates the distribution of PS lifetimes when all PS are properly sampled from an epoch of fibrillation. **(D)** Demonstrates the distribution of PS lifetimes when only PS present at a randomly chosen timepoint (such as the commencement of a recording) are observed.

An overview of the computational models, fibrillation data used, and analytical approach is provided in Figure 2.

#### Computational models

##### Aliev-Panfilov model

A simulation of fibrillation-like spiral-defect chaos was generated using the APV model (Aliev and Panfilov, 1996). In this a spiral wave was generated on an map with an inhomogeneous surface, leading to break up and additional spiral waves and free wavelets. A 200 × 200-pixel simulation was computed. Ten simulations were developed using identical model parameters, but with different patterns of randomly distributed inhomogeneities, $\mu_{1}$ . A full description of the model details are provided in Supplementary Material S2.

##### Atrial fibrillation models

Computational models of AF were also used to examine the influence of an inspection paradox. The computational models of AF were based on the Courtemanche-Ramirez-Nattel cell model (Courtemanche et al., 1998; Kneller et al., 2002; Aguilar et al., 2017) These models were developed as described during a previously published study (Dharmaprani et al., 2021) The full details of these models are further detailed in Supplementary Material S3.

#### Animal models of fibrillation

Optical mapping was performed as previously described in rat AF and VF models (Aguilar et al., 2015; Xiong et al., 2015; Nattel et al., 2017; Dharmaprani et al., 2019b).

For AF, the heart was excised and perfused with Krebs solution at 30 ml/min and 37°C for 30 min. Following electrical/mechanical decoupling with blebbistatin (15 μmol/L), the heart was loaded with di-4-ANEPPS (Biotium, Inc, Hayward, CA). RA free wall fluorescence was recorded at 1 kHz, using a charge coupled device (CardioCCD, RedShirtImaging, LLC, Decatur, Georgia). Recording duration was 1 s for *n* = 3 cases.

In VF, rat hearts were retrogradely perfused with oxygenated Tyrodes solution (37°C, perfusion pressure 60 mmHg). Recording duration was 2 s for *n* = 10 cases (Ilkan et al., 2018; Dharmaprani et al., 2019b; Strauss et al., 2019).

#### Human fibrillation mapping

The study also included data from human AF and VF. Human AF data was mapped from previously analysed basket catheter and HD-grid recordings prior to AF ablation (Dharmaprani et al., 2019a; Dharmaprani et al., 2019b; Dharmaprani et al., 2021; Schopp et al., 2021) For the basket data, 64-electrode basket catheters were used [Constellation, Boston Scientific, 48 mm (4 mm spacing), 60 mm (5 mm spacing)]. Unipolar electrogram recordings were obtained from patient recordings [0.5–500 Hz, 2000 Hz sampling frequency]. For basket catheter recordings, *n* = 11 patients for the basket catheters (mean duration = 56.54 s, 95% CI = 1.74 s), and for HD-grid recordings *n* = 10 patients. Ethics approval was obtained (IRB approval number 110634). For HD-grid data, a 16-pole Advisor™-HD grid catheter (3 mm electrodes, 3 mm equidistant spacing) was used to obtain 1-min recordings from the left inferior pulmonary vein.

The human VF data utilised in this study was acquired as previously described (Nash et al., 2006) In brief, this data was obtained from patients undergoing cardiac surgery. A 256-electrode epicardial sock was placed around the ventricle, and VF was induced by electrical stimulation after cross-clamping the aorta. 2.5-min unipolar electrogram signals were recorded [1000Hz sampling frequency] with a UneMap recording system. Ethics approval was obtained (IRB approval number REC 01/0130).

#### Signal processing and phase singularity detection

Signal pre-processing was performed as in previous studies (Nash et al., 2006; Dharmaprani et al., 2019b; Dharmaprani et al., 2021). For human AF recordings, the unipolar electrograms and surface electrograms were exported from NavX. QRS subtraction was performed, followed by 4th order 1–30 Hz bandpass Butterworth filtering, 8th order 10 Hz lowpass Butterworth filtering, and sinusoidal recompositioning (Kuklik et al., 2014; Dharmaprani et al., 2021). For human VF unipolar epicardial signals from the 256 electrode epicardial sock were mapped onto a 2D grid, with the instantaneous phase calculated using the Hilbert Transform (Nash et al., 2006). Instantaneous phase was calculated by applying the Hilbert transform to sinusoidally reconstructed signals (Dharmaprani et al., 2021; Dharmaprani et al., 2022). PS tracking was performed as previously described, utilizing an algorithm to compare the locations of PS in order to create a continuous data-table of when a new PS occurs and when it has been destroyed (Dharmaprani et al., 2019b; Dharmaprani et al., 2021; Dharmaprani et al., 2022). This data-table was used to construct a distribution of PS lifetimes. This distribution was fit to an exponential distribution using maximum likelihood as previously described (Dharmaprani et al., 2021; Dharmaprani et al., 2022). Signal processing and PS detection techniques are further described in Supplementary Material S4 and S5

#### Simulating observation/recording at random times

To model the effect of observing an inspection paradox, 10,000 randomly selected commencement timepoints were selected across full recordings of fibrillation. These timepoints represent an instance of observation and demonstrate the potential impact of short-term observations around these points. The start times and end times of all PS active at the selected timepoints were used to create a list of lifetimes. The means of the overall PS lifetimes and the 10,000 samples were compared.

#### Statistical analysis

Distributional fitting was performed using maximum likelihood estimation. Distributional parameters were compared with a paired sample t-test, with significance α-value set at *p* = 0.05. An important consideration in the computational simulations is that of *p*-values, due to the fact that these can be replicated many times (Liberos et al., 2017). In the interests of interpretability to a wider scientific audience, we have presented findings with *p*-values and specified the number of replicates.

#### Sensitivity analysis

A sensitivity analysis was performed in order to confirm that the different statistics between the observed vs. overall PS lifetime distributions were due to an inspection paradox. To address this several approaches were taken, including 1) adjustment of grid sizes in the simulated data to confirm that the size of the observed field of view did not induce an inspection paradox, 2) use of a 3D model of fibrillation to confirm that it is not a feature of 2D mapping techniques.

For the *n* = 10 simulations generated using the Aliev-Panfilov computational simulations, the grid size was varied. The grid size was varied from 50 × 50 pixels to 200 × 200 pixels. This was performed through constraining the 200 × 200 grid, to 150 × 150 pixels, 100 × 100 pixels, 80 × 80 pixels, 50 × 50 pixels, 30 × 30 pixels, and 20 × 20 pixels.

A 3D computational simulation of AF using the Courtmanche-Ramirez-Nattel model was also examined (Courtemanche et al., 1998). The model was constructed from cardiac MRI data of an AF patient. This model was generated during a previously published study (Roney et al., 2020).

## Results

We examined the potential for an inspection paradox in seven model systems: 1) two computational models; 2) two experimental models of AF and VF examined with optical mapping; and 3) three mapping modalities for human AF and VF data.

### Computational simulations

**FIGURE 3:**
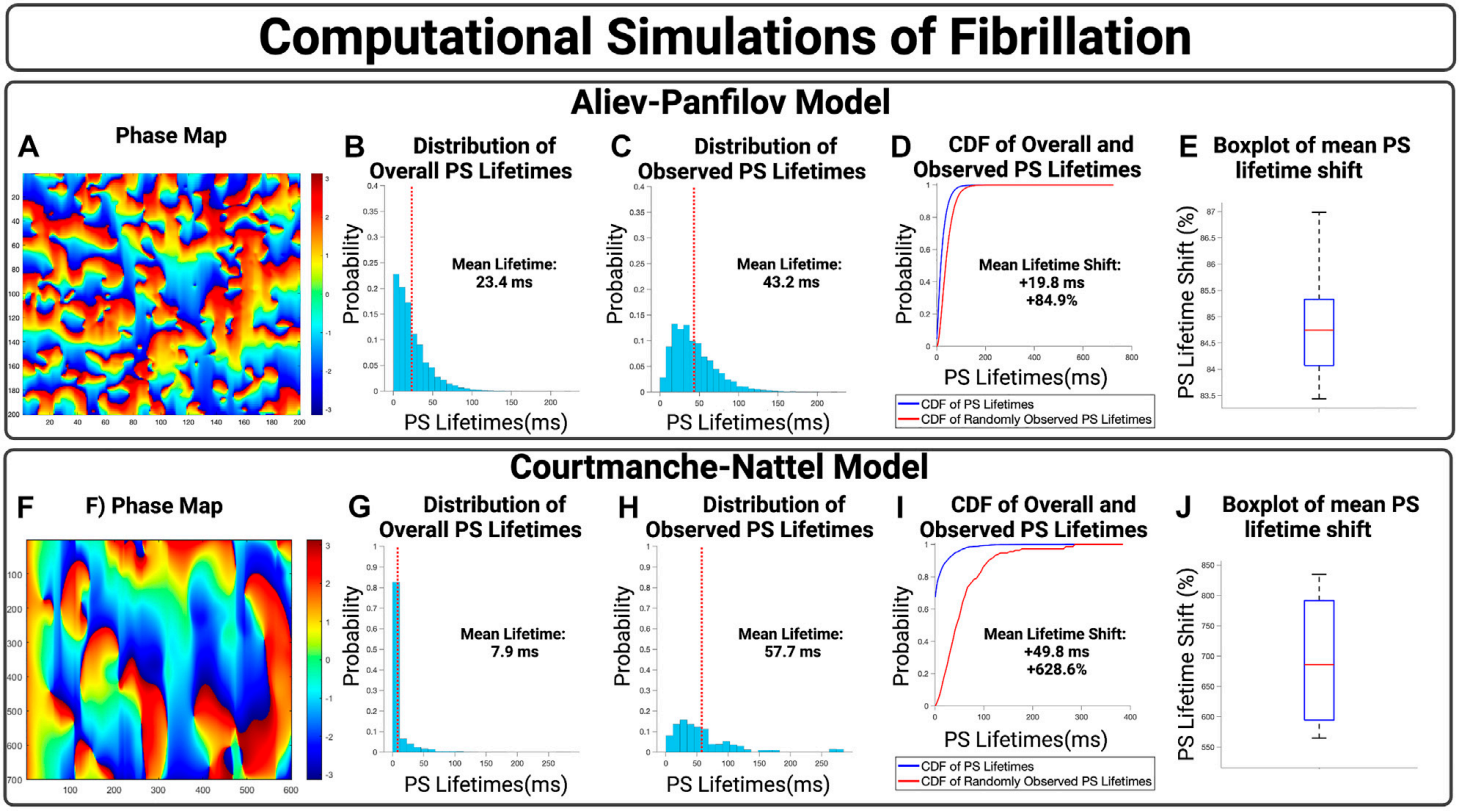
**(A–D)** Presents an example case of an APV simulation. **(B,G)** presents the overall distribution of PS lifetimes across a full epoch of their respective model systems. **(C,H)** Demonstrate the distribution of observed PS sampled at the 10,000 commencement timepoints. **(D,I)** Presents the cumulative distribution functions (CDF) of the overall and observed PS lifetimes. **(E)** shows the boxplot of mean lifetime shifts across n = 10 APV cases, with an increase in the mean PS lifetime of +19.7 ms (95% CI, ± 0.1 ms) and an overall lifetime shift of +84.9% (95% CI, ± 0.3%). (*p* < 0.001 for observed vs. overall). **(F–I)** presents an example case of a 2D computational model of fibrillation. **(J)** For n = 4 cases, there was an increase in mean PS lifetimes of +45.5 (95% CI, ± 1.9 ms) and an overall lifetime shift of +692.9% (95% CI, ± 57.7%). (*p* < 0.001 for observed vs. overall).

We first show the effect of an inspection paradox in the APV model, Figures 3A–E. In Figures 3A–D, an example case is presented with an observed mean PS lifetime increase of +59.8 ms and overall lifetime shift of +115.3%. For *n* = 10 cases, the mean increase in PS lifetimes +19.7 ms (95% CI, ± 0.1 ms) and an overall mean lifetime shift of +84.9% (95% CI, ± 0.3%) (*p* < 0.001 for observed vs. overall).

The effect of an inspection paradox is next shown in 2D AF simulations are shown in Figures 3F–J. In Figures 3F–I, an example case is presented with an increase in mean PS lifetime of +49.8 ms and an overall lifetime shift of +628.6%. For *n* = 4 cases, there was an increase in mean PS lifetimes of +45.9 ms (95% CI, ± 1.9 ms) and a mean PS lifetime shift of +692.9% (95% CI, ± 57.7%) (*p* < 0.001 for observed vs. overall).

### Atrial fibrillation

**FIGURE 4:**
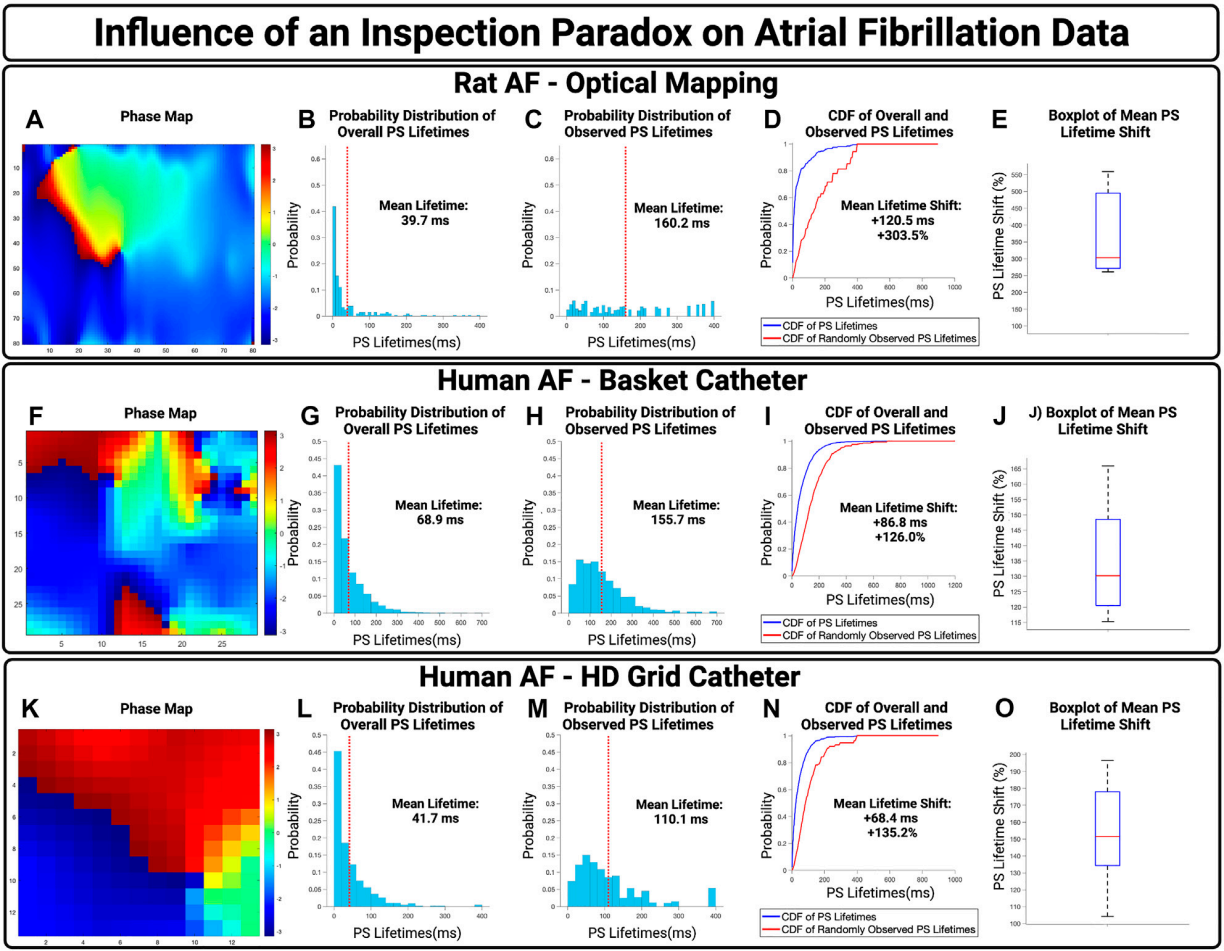
The influence of an inspection paradox on AF data. **(A–D)** Presents an example case of optically mapped rat AF, **(F–I)** presents an example case of basket catheter recorded human AF, **(K–N)** Presents an example case of HD grid recorded human AF. **(B,G,L)** Presents the overall distribution of PS lifetimes across full epochs of AF. **(C,H,M)** Demonstrates the distribution of observed PS sampled at the 10,000 commencement timepoints. **(D,I,M)** Presents the cumulative distribution functions (CDF) of the overall and observed PS lifetimes. **(E)** In n = 3 cases of optical mapping, the increase in mean PS lifetime was 122.6 ms (95% CI, ± 27.5 ms) and the overall mean PS lifetime shift was +374.6% (95% CI, ± 88.5%) (*p* = 0.052 for observed vs. overall). **(J)** In n = 10 cases, the increase in mean PS lifetime was 78.8 ms (95% CI, ± 9.2 ms) and the overall mean lifetime shift of PS was +129.2% (95% CI, ± 4.1%) (*p* = 0.04 for observed vs. overall). **(O)** In n = 10 cases, the increase in mean PS lifetime was 64.3 ms (95% CI, ± 5.8 ms) and the overall mean PS lifetime shift was +150.8 (95% CI, ± 9.0%) (*p* < 0.001 for observed vs. overall).

To examine the potential influence of an inspection paradox in experimentally acquired AF, we first examined optically mapped AF in rat models. A representative case is presented in Figures 4A–D, with a PS lifetime increase of +120.5 ms and a PS lifetime shift of +303.5%. For *n* = 3 cases, the mean increase in observed PS lifetimes was +122.6 ms (95% CI, ±  27.5 ms) and a mean PS lifetime shift was +374.6 (95% CI, ±  88.5%) (*p* = 0.052 for observed vs. overall).

The potential effect of an inspection paradox was next examined in human AF. For basket catheter recordings, a representative example case is shown in Figures 4F–I, with an observed PS lifetime increase of +86.6 ms and a PS lifetime shift of +126.0%. For *n* = 10 cases, the mean increase in PS lifetimes was 82.6 ms (95% CI, ±  7.1 ms) and a mean PS lifetimes shift of 135.3% (95% CI, ±  5.0%) (*p* < 0.04 for observed vs. overall).

Human AF recorded using HD Grid Catheters was also examined. From a sample of *n* = 10, a representative example is presented in Figure 4K–N, with an increase in mean observed PS lifetimes of +68.4 ms and an overall PS lifetime shift of +135.2%. For *n* = 10 cases, the mean increase in PS lifetimes was +64.3 ms (95% CI, ±  5.8 ms) and a mean PS lifetime shift of +150.8% (95% CI, ±  9.0%) (*p* < 0.001 for observed vs. overall).

In basket catheter, HD-grid catheter, and optical recordings of AF, the probability distributions of observed PS lifetimes were shifted towards a higher probability of longer lifetimes.

### Ventricular fibrillation

**FIGURE 5:**
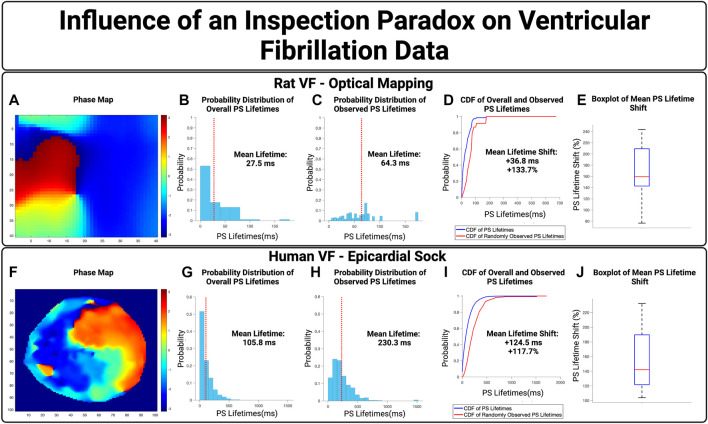
The Influence of an inspection paradox on VF data. **(A–D)** Show an example case of optically mapped rat VF, and **(F–I)** shows an example case of human VF recording through a 256 electrode epicardial sock. **(B,G)** Show the overall distribution of PS lifetimes across full epochs of VF. **(C,H)** Show the distribution of PS lifetimes observed across the 10,000 samples. **(D,I)** Presents the cumulative distribution functions (CDF) of the overall and observed PS lifetimes. **(E)** In optically mapped rat VF, there was an increase in the mean PS lifetime of +40.0 ms (95% CI, ±5.1 ms) and an overall mean lifetime shift of +171.3% (95% CI, ± 15.6%) (*p* < 0.001 for observed vs. overall). **(J)** In human VF, in n = 8 cases, there was an increase in the mean PS lifetime of +159.5 ms (95% CI, ±15.2 ms) and an overall mean lifetime shift of +153.5% (95% CI, ± 15.7%) (*p* < 0.001 for observed vs. overall).

We next examined the influence of an inspection paradox in VF data. Optically mapped VF from rat models were first used, with an example case in Figures 5A–D. This example demonstrated a mean PS lifetime increase of +36.8 ms and an overall lifetime shift of +133.7% Across *n* = 10 cases, there was an increase in the mean PS lifetime of +40.0 ms (95% CI, ± 5.1 ms) and an overall shift in the mean PS lifetime shift of +171.3% (95% CI, ± 15.6%) (*p* < 0.001 for observed vs. overall).

We next evaluated the potential effect of an inspection paradox in human VF. A representative example is shown in Figures 5F–I, from patients with human VF, As summary box plot is shown in Figure 5D showing the range of mean PS lifetime shifts. In *n* = 8 cases examined, the increase in mean lifetime of PS was +159.5 ms (95% CI, ± 15.2 ms) and an overall shift in the mean lifetimes of PS +153.5% (95% CI, ± 15.7%) (*p* < 0.001 for observed vs. overall).

Overall, in both epicardial electrogram recordings of human VF and optically mapped rat VF, the probability distributions of observed PS lifetimes were shifted towards a higher probability of longer lifetimes.

### Sensitivity analysis

As part of a sensitivity analysis, a 3D computational simulation of fibrillation in a detailed geometry was also examined. In this simulation, an inspection paradox could result in an increase in observed PS lifetimes of +791.7 ms and a mean lifetime shift of +691.7%.

An additional sensitivity analysis was performed examining whether the size of the system influenced the computational simulations. At different grid sizes, an inspection paradox led to increases in the mean lifetime observed. At 20 × 20 pixels a relative lifetime increase of +114.8% (+17.2 ms), at 30 × 30 pixels a relative increase of +105.2% (+18.0 ms), at 50 × 50 pixels a relative increase of +94.1% (+18.4 ms), at 80 × 80 pixels a relative increase of +87.9% (+18.6 ms), at 100 × 100 pixels a relative increase of +87.1% (+18.9 ms), at 150 × 150 = pixels a relative increase of +85.5% (+19.3 ms), at 200 × 200 pixels a relative increase of +84.9% (+19.7 ms).

## Discussion

Spiral vortex regeneration is a feature of many spatiotemporally turbulent systems in nature. The statistical properties of topological defects known as phase singularities (PS), located at the pivot of spiral wave vortices, appear to be common, despite differences in underlying generating processes. Specifically, the populations of PS have been shown to adopt Poisson-type distributions, (Gil et al., 1990; Wang, 2004) and the lifetimes of PS appear to be exponentially distributed (Tan et al., 2020). These statistical properties are important because they are consistent with a notion of effective statistical independence of creation and annihilation events.

It appears that cardiac fibrillation may share these statistical properties. A substantial body of evidence from multiple laboratories using multiple mapping approaches in AF and VF suggests that lifetimes of spiral vortices in AF and VF (and the more sustained spirals lasting more than one rotation) also adopt exponential-type distributions (Chen et al., 2000a; Chen et al., 2000b; Kay et al., 2006; Kuklik et al., 2016; Child et al., 2018; Christoph et al., 2018). We recently confirmed these data, with these studies of AF and VF suggesting the population dynamics of spirals in cardiac fibrillation analogous to spiral vortex regeneration in physical, chemical and biological systems in nature (Dharmaprani et al., 2019b; Dharmaprani et al., 2021; Dharmaprani et al., 2022).

Cardiac fibrillation is characterised by continuous regeneration of spiral waves (Dharmaprani et al., 2019b). Recently, we have shown that PS and wavelet regeneration can be modelled as renewal processes (Dharmaprani et al., 2019b; Dharmaprani et al., 2021). In a renewal process, individual events are effectively statistically independent, and over time may converge to a constant hazard rate (Ross, 2014). We have shown that the formation and destruction of PS and wavelets may adopt this property in human, experimental and simulated cardiac fibrillation (Dharmaprani et al., 2019b; Dharmaprani et al., 2021). The renewal theory approach has a number of key strengths: 1) It provides simple probabilistic models to analysing clinical and experimental arrhythmias; (Qu, 2022) 2) It connects fibrillatory dynamics to other spatiotemporally turbulent natural systems characterised by regeneration of spiral vortices—providing useful principles that may assist understanding challenging problems in AF and VF, such as the difficulty in reliable identification of driving rotors, and the origin of the critical mass hypothesis (Zipes et al., 1975; Kim et al., 1997; Vidmar and Rappel, 2019).

In this study, we sought to evaluate the impact of the renewal process on PS lifetime observation. We hypothesised that if PS lifetimes are modelled as renewal processes, there was a possibility that PS occurring around the commencement of a recording would have disproportionately longer lifetimes than the true mean PS lifetime, due to an inspection paradox. In periods of short observation, such as short recordings during optical mapping studies, there would a relative increase in the probability of oversampling prolonged PS lifetimes, in particular at the initiation of a recording.

We illustrated that the inspection paradox could lead to a relative emphasis on prolonged events in a theoretical simulated renewal process. To date, the possibility of an inspection paradox in recordings of cardiac fibrillation has not to our knowledge previously been established in prior published investigations. A strength of our study is the comprehensive approach to validation and quantitation of this effect in multiple settings. Specifically, we have shown that an inspection paradox has the potential to occur in both AF and VF, in simulated, and experimentally mapped AF and VF in animal models and humans, and that it was independent of the modality by which cardiac fibrillation is recorded. This suggests that the possibility of inspection paradoxes is a general property of fibrillatory dynamics that should be considered in both mechanistic and clinical studies of AF and VF.

The potential influence of an inspection paradox is particularly noticeable in systems which have not undergone long periods of recording, such as the optical mapping datasets utilized here. The distributions of observed lifetimes lack the distinctive shape present in systems with longer recordings (such as the basket catheter, HD-grid catheter, epicardial VF sock, and computational simulations utilized in this study), while still demonstrating that longer lifetime events are more probably to occur, as shown by the greater mean lifetime for observed PS lifetimes. This suggests that an inspection paradox can have a more distorting influence on shorter datasets, with a preference for long lifetime PS.

An inspection paradox is a potentially important consideration in efforts to ablate re-entrant circuits as potential “drivers” of AF, because purely visual inspection of AF recordings would tend to fall during periods of relatively sustained re-entry. It would therefore be important to examine the overall temporal distribution of re-entrant circuit lifetimes to avoid inadvertent over-sampling of longer lasting PS by chance.

### Relationship to spatiotemporal turbulence in other natural systems

A fundamental property of nonequilibrium spatiotemporally turbulent systems throughout nature is the presence of the regeneration of spiral wave type vortices (Cross and Hohenberg, 1993; Cross and Greenside, 2009). This property of repetitive creation and annihilation of spiral defects have been shown to have common statistical laws, with an exponential distribution of defect lifetimes and Poisson distribution of PS population (Tan et al., 2020; Jenkins et al., 2022). These properties have been shown to apply in a variety of physical (Morris et al., 1993; Ouyang and Flesselles, 1996; Egolf et al., 2000), chemical (Beta et al., 2006; Qiao et al., 2009), and biological systems (Lechleiter et al., 1991; Tan et al., 2020) characterised by the repetitive creation and destruction spirals. These properties have recently been shown to apply to the comparably spatiotemporally turbulent biological process of cardiac fibrillation in the heart (Dharmaprani et al., 2019b; Dharmaprani et al., 2021; Dharmaprani et al., 2022). The consistency of these statistical properties is evident in the visual distributions of PS lifetimes in cardiac fibrillation published by multiple laboratories around the world (Chen et al., 2000a; Chen et al., 2000b; Kay et al., 2006; Kuklik et al., 2016; Child et al., 2018; Christoph et al., 2018).

Given the consistency of these statistical laws across spatiotemporally turbulent systems in nature, their validation in the context of cardiac fibrillation is to be expected. Further, it would strongly suggest that the distributions of PS defect lifetimes observed in this study, and previous investigations of cardiac fibrillation, have not occurred due to the method of sampling or mapping, but are a fundamental and generic property of the spatiotemporal turbulence as a natural phenomenon. We have recently shown that the origin of these statistical properties arises in the effective statistical independence of consecutive formation and destruction events (Jenkins et al., 2022).

The current manuscript deals the inspection paradox in the context of cardiac fibrillation as an example of natural biological spatiotemporal turbulence, However, given the common statistical properties of topological defect dynamics in cardiac fibrillation and other systems in nature, the issue of an inspection paradox has the potential to have a broader occurrence in each of these other systems.

### How to avoid the inspection paradox

The purpose of the current manuscript is to highlight the importance of considering the possibility of an inspection paradox arising in recordings of AF and VF. The keys to avoiding the inspection paradox are: 1) Focus on the overall distribution of PS lifetimes as opposed to the most-sustained PS lifetimes; 2) As far as possible use continuous recording modalities (e.g., with dyes that are resistant to photobleaching, or continuous electrogram recording), to enable the full distribution of PS lengths to be fully characterised. To understand the fibrillatory process, it may require a focus on the lifetimes of all lengths of PS, rather than consideration of only the longest re-entrant episodes. We would suggest in publications that examine PS lifetimes the overall PS lifetime distribution should be published, ideally not just the longest lasting re-entry episodes. If possible, renewal rate constants for the rate of formation and destruction of re-entrant circuits should be examined, as these metrics have the potential to be more statistically stable than the lifetime of individual re-entrant circuits (Dharmaprani et al., 2019b; Dharmaprani et al., 2021; Dharmaprani et al., 2022).

## Conclusion

The inspection paradox can be a potential source of inadvertent error when observing AF or VF. Visual inspection of phase movies has the potential to systematically oversample prolonged longer-lasting PS, giving the impression of sustained rotors. The effect of the inspection paradox is minimised by careful consideration of the overall PS lifetime distribution over prolonged windows in cardiac fibrillation recordings.

## Data Availability

The original contributions presented in the study are included in the article/Supplementary Material, further inquiries about the data used to support conclusions of this article can be directed to the corresponding author.

## Abbreviations

PS, Phase Singularities; AF, Atrial Fibrillation; VF, Ventricular Fibrillation; APV model, Aliev-Panfilov model.
