# Supplementary material for: The inspection paradox: An important consideration in the evaluation of rotor lifetimes in cardiac fibrillation
Source: Front Physiol. 2022 Sep 6;13:920788. doi: 10.3389/fphys.2022.920788 (PMC9486478; doi:10.3389/fphys.2022.920788)
Supplement: Supplementary file 1 [file DataSheet1.DOCX]

**Supplemental Materials**

**The inspection paradox: an important consideration in the evaluation of rotor lifetimes in cardiac fibrillation**

**S1 MATLAB Code to Illustrate the Effect of an Inspection Paradox**

To demonstrate effect of an inspection paradox, a simple simulation of phase singularity formation and destruction was simulated, defined by $\lambda_{f}$ (formation) and $\lambda_{d}$ (destruction). These were used to develop two probability distributions, from which PS inter-formation timings were drawn from the one defined by $\lambda_{f}$, and the subsequent destruction time was defined by random draws from the probability distribution defined by $\lambda_{d}$. In the following code, partially adapted to MATLAB from Python code developed by Ravinatula,(Ravinutala, 2018) we demonstrate how inspection paradoxes may occur in a renewal process.

clear

clc

close all

num_samps = 1000; %how many events(PS/WF) should be simulated

num_simulations = 1000; % how many simulations should be made

roundUp = true; %should values be rounded or should decimals be allowed.

fs = 2000; %Sampling frequency

lambda_f = 0.012; %Formation rate In 1/ms

mu_f = 1/lambda_f;

lambda_d = 0.009; %Destruction Rate

mu_d = 1/lambda_d;

nbins = 15;

pd_f = makedist('Exponential', mu_f);

pd_d = makedist('Exponential', mu_d);

[sim_formation_times, wait_times]= simulate_PS_formation(num_samps, num_simulations, pd_f, roundUp);

[sim_destruction_time, sim_Lifetimes] = simulate_PS_lifetimes(sim_formation_times, num_samps, num_simulations,pd_d, roundUp);

X = randi([0,round(max(max(sim_formation_times)/1.1))], 1,1);

X = X - 0.5;

formations_around_strike = zeros(num_simulations,1);

for iteration = 1:num_simulations

formations_around_strike(iteration) = get_time_window(sim_formation_times(iteration,:),X);

end

for iteration = 1:num_simulations

formations_after_strike(iteration) = get_time_to_next(sim_formation_times(iteration,:),X);

end

for iteration = 1:num_simulations

temp{iteration}= get_lifetimes_present(sim_formation_times(iteration,:),sim_destruction_time(iteration,:), sim_Lifetimes(iteration,:),X);

end

lifetimes_during_window = cell2mat(temp);

%% Formation Times

figure

subplot(2,1,1)

histogram(wait_times,nbins,'Normalization','probability')

meanWaitArray = mean(wait_times,2,'omitnan');

meanWait = mean(meanWaitArray);

title(sprintf('Mean Formation time: %d', round(meanWait,0)))

ylabel('Probability')

xlabel('Formation Times / Interevent Times')

subplot(2,1,2)

histogram(formations_around_strike,nbins,'Normalization','probability')

mean_formations_Window = mean(formations_around_strike,'omitnan');

title(sprintf('Mean Formation around strike time: %d',round(mean_formations_Window,0)))

ylabel('Probability')

xlabel('Formation Times / Interevent Times')

%%

waitDis = fitdist(meanWaitArray, 'Poisson');

yWait = pdf(waitDis, 1:150);

yWaitCDF = cumsum(yWait);

figure

plot(yWaitCDF, 'b', 'linewidth', 3,'Linestyle',':')

ylim([0 1.05])

title('CDF')

hold on

text(meanWait, 0.5, sprintf('%d', round(meanWait)));

windowDis = fitdist(formations_around_strike, 'Poisson');

yWindow = pdf(windowDis, 1:150);

yWindowCDF = cumsum(yWindow);

plot(yWindowCDF,'r','linewidth', 3,'Linestyle','--')

text(mean_formations_Window, 0.5, sprintf('%d', round(mean_formations_Window)));

hold off

%% Calculate lambda F

if size(wait_times,1) > 1

pd_f_all = fitdist(wait_times(2,:)', 'exponential'); % Fit to exponential

else

pd_f_all = fitdist(wait_times', 'exponential'); % Fit to exponential

end

sim_lambda_f = 1/pd_f_all.mu;

%% Lifetimes

figure

h = histogram(sim_Lifetimes,nbins,'Normalization','probability', 'EdgeAlpha',0.5, 'FaceColor', [0.03010 0.7450 0.9330], 'FaceAlpha', 0.9);

yLimit = h.Values(1) + 0.02;

meanLifetimeArray = mean(sim_Lifetimes,2,'omitnan');

meanLifetime = mean(meanLifetimeArray);

hold on

plot([meanLifetime meanLifetime], 0:1,'r', 'LineStyle', ':', 'linewidth', 5)

hold off

ylim([0 yLimit])

ylabel('Probability')

xlabel('Lifetime (ms)')

set(gca, 'FontName', 'Helvetica');

set(gca, ...

'Box' , 'off' , ...

'TickDir' , 'out' , ...

'TickLength' , [.02 .02] , ...

'XMinorTick' , 'off' , ...

'YMinorTick' , 'off' , ...

'XGrid' , 'off' , ...

'YGrid' , 'off' , ...

'XColor' , [.3 .3 .3], ...

'YColor' , [.3 .3 .3], ...

'LineWidth' , 1 );

set( gca,'FontName', 'Helvetica' );

set(gca,'FontSize' ,20);

fig = gcf;

fig.PaperUnits = 'inches';

fig.PaperPosition = [0 0 9 9];

print('Inspection Paradox True Distribution','-dpng','-r0')

figure

histogram(lifetimes_during_window,nbins,'Normalization','probability', 'EdgeAlpha',0.5, 'FaceColor', [0.03010 0.7450 0.9330], 'FaceAlpha', 0.9)

mean_lifetimes_Window = mean(lifetimes_during_window,'omitnan');

hold on

plot([mean_lifetimes_Window mean_lifetimes_Window], 0:1,'r','LineStyle', ':', 'linewidth', 5)

hold off

ylim([0 yLimit])

ylabel('Probability')

xlabel('Lifetime (ms)')

set(gca, 'FontName', 'Helvetica');

set(gca, ...

'Box' , 'off' , ...

'TickDir' , 'out' , ...

'TickLength' , [.02 .02] , ...

'XMinorTick' , 'off' , ...

'YMinorTick' , 'off' , ...

'XGrid' , 'off' , ...

'YGrid' , 'off' , ...

'XColor' , [.3 .3 .3], ...

'YColor' , [.3 .3 .3], ...

'LineWidth' , 1 );

set( gca,'FontName', 'Helvetica' );

set(gca,'FontSize' ,20);

fig = gcf;

fig.PaperUnits = 'inches';

fig.PaperPosition = [0 0 9 9];

print('Inspection Paradox Observed Distribution','-dpng','-r0')

if size(sim_Lifetimes,1) > 1

pd_d_all = fitdist(sim_Lifetimes(2,:)', 'exponential'); % Fit to exponential

else

pd_d_all = fitdist(sim_Lifetimes', 'exponential'); % Fit to exponential

end

sim_lambda_d = 1/pd_d_all.mu;

save('inspection_paradox_sim_data.mat')

**S2 Aliev-Panfilov model of cardiac fibrillation**

An Aliev-Panfilov model was used to simulate fibrillatory activity.(Aliev and Panfilov, 1996) We used a rectangular grid of 200x200 diffusively coupled simulation nodes (diffusion coefficient set to 0.2). Space step was set to 0.6 and time step to 0.02. The system was integrated using a forward Euler scheme with no-flux boundary conditions. In order to model an inhomogeneous substrate for AF we varied parameter μ2 in the model which strongly influences steepness of the restitution curve. We introduced a random distribution of μ2 which led to the presence of localized areas of high dynamical instability as previously described. Based on a study by ten Tusscher and Panfilov, we used following parameters of the model: a=0.1, k=8, ε=0.01, b=0.1, μ2=0.2.(Ten Tusscher and Panfilov, 2003) In order to induce dynamical instability, we varied the distribution of μ1 from 0.02 and 0.2. This was repeated 10 times, to produce simulations with different distributions of μ1.

**S3 - Computer Simulation of Atrial Fibrillation – Courtmanche-Ramirez-Nattel Model**

The Courtemanche model of AF used here was adapted from one previously described.(Aguilar et al., 2017) The Courtemanche model of the human atrial cardiomyocyte was used,(Courtemanche et al., 1998) that was adapted and implemented in as a monodomain model in CARP, running in a parallel cloud-based cluster. Tissue conductance was set to provide physiological anisotropy with conduction velocity in a longitudinal direction of 47.9 cm/s. The tissue slab was 7x6cm in size, with longitudinal fiber anisotropy with grid ration of 6. Grid discretization was performed at 100µm resolution. The membrane capacitance was set to 1µF/cm.(Courtemanche et al., 1998) Differential equations were solved with a 25µs timestep, with simulations of up to 5 seconds. Atrial fibrillation epochs were initiated by a standard S1-S2 cross-shock protocol. 2D simulations were performed with a ACh distribution was generated by randomly assigning a value between 0 and 0.001 μM to each node (cardiomyocyte). The ACh-activated K+ current was implemented using the previously-published model by Kneller et al.(Kneller et al., 2002)

# S4: Data Processing Techniques

## **4a. Atrial Fibrillation**

Baseline drift was removed from signals by removing the best straight-fit line (detrending). A template subtraction method was used to remove far field ventricular depolarisation as described previously(Shkurovich et al., 1998), following baseline correction of each epoch. Specifically, the template subtraction method identifies fiducial points for ventricular complexes using the QRS detection algorithm by Pan and Tompkins(Pan and Tompkins, 1985). An average or ‘median’ ventricular complex was then constructed by aligning the detected ventricular complexes at their respective fiducial points, and performing a median operation of the matching points in all complexes (Shkurovich et al., 1998). Subtracting the median complex from each ventricular complex resulted in QRS subtracted electrograms. Further pre-processing was applied using a 3rd order Butterworth fitted with a 40-250 Hz band pass filter, and an 8^th^ order Butterworth filter fitted with 10 Hz low pass filter applied in forward and reverse mode.(Roney et al., 2017b; Dharmaprani et al., 2019)

Following this, sinusoidal recomposition of the signal was applied to transform the AF electrogram signal into a sum of sinusoidal wavelets. Doing so increases the accuracy of the phase reconstruction using the Hilbert transform, as mathematically this transformation works best on signals with a sinusoidal morphology (e.g. ventricular signals). As atrial signals are characterised by long iso-electric intervals between consecutive deflections, these can result in artifacts in the reconstructed phase. The sinusoidal recomposition transforms AF electrograms into sinusoidal wavelets which only exist when there is a negative derivative in the original signal (as a negative slope in a unipolar electrogram corresponds to the passing of a wavefront). The amplitude of the wavelet is proportional to the slope of the signal, and has a period equal to the mean cycle length of the electrogram (which was derived from the dominant frequency of the electrogram). These properties result in more accurate phase reconstruction for subsequent PS analyses.

## **4b. Ventricular Fibrillation**

To allow for phase and wavefront detection, electrode coordinates were projected on a 2D plane. The three-dimensional co-ordinates of mesh vertices were mapped onto a 2D polar plot using a cone-shaped surface projection and Delaunay triangulation.(Nash et al., 2006b) Using this 2D projection, electrode potentials were linearly interpolated from the electrodes onto a fine regular grid (100x100 grid points).

To remove electrodes possessing poor signal-to-noise ratios, these electrograms were removed from the analyses prior to 2D projection of the mesh by selecting only signals with a dominant frequency within the 1.5 to 45 Hz band for analyses. (Nash et al., 2006b) EGMs near the stimulus electrode at times also demonstrated an exponential artifact, as well as respiration artifacts. To remove this, a mode was fitted with a constant offset of the signal mean, an exponential term, as well as a sinusoidal term. This fitted signal was subtracted from the raw signal to improve signal to noise ratio.(Nash et al., 2006b)

# S5: Hilbert Transform and Phase Singularity Detection

## **5a. Hilbert Transform and Phase**

In the context of cardiology, phase can be used to study electrical wave propagation. In this study, the instantaneous phase for each electrogram was reconstructed by applying the Hilbert transform on the cleaned and sinusoidally reconstructed signal for intracardiac unipolar EGM(Bray and Wikswo, 2002b; Kuklik et al., 2015), and using the transmembrane voltage for computer simulated fibrillation. Mathematically, the Hilbert transform can be given as follows:

$$\phi\left( t \right)=arctan\left( \frac{-u\left( t \right)-u*}{H\left( u \right)\left( t \right)-u*} \right)$$

where $u*$ sets the origin of the phase plane with respect to the phase that is computed. (Kuklik et al., 2015) After applying the Hilbert transform, the instantaneous phase was interpolated using complex vector interpolation to avoid incorrect phase calculation. (Roney et al., 2017a; Vidmar and Rappel, 2018)

Plotting the phase values at each recorded site of the heart for all time points *t* results in phase maps such as those shown in Figure 1a, 1F, and 1K. Phase is colour coded, allowing visual interpretation of the propagation of electrical activity.


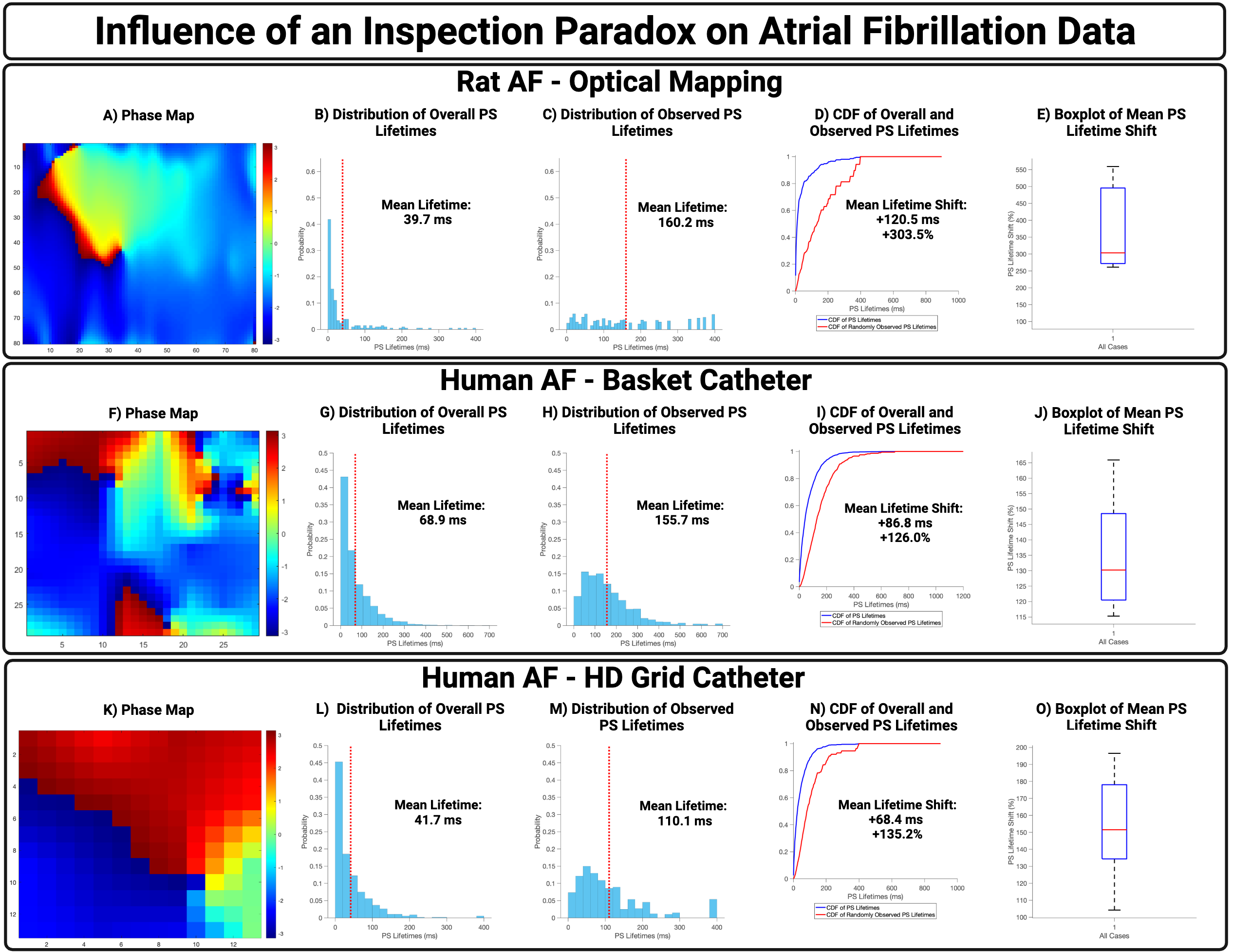


Figure 1: Taken from the manuscript (Figure 4)

## **5b. PS Detection and Tracking**

In this study, we use a convolution kernel method(Bray and Wikswo, 2002a; Nash et al., 2006a) to detect PS. The convolution kernel method approximates the gradient of phase from the discretized phase map using a finite difference operation in the $x$ and $y$ directions, given by:

$k_{x}\left[ m,n \right]= \nabla_{\phi_{x}}\left[ m,n \right]= \phi\left[ m+1, n \right]- \phi\left[ m, n \right]$

$k_{y}\left[ m,n \right]= \nabla_{\phi_{y}}\left[ m,n \right]= \phi\left[ m, n+1 \right]- \phi\left[ m, n \right]$

where is $\phi$ phase, and $\left[ m,n \right]$ the pixel coordinates. The line integral at the pixel $\left[ m,n \right]$ can there be approximated using the convolution kernel equation:

$$\left( \nabla X\vec{k} \right)\cdot\hat{z}\propto\nabla_{x}\otimes k_{y}+\nabla_{y}\otimes k_{x}$$

where $\otimes$ is the convolution operator, and $\nabla_{x}$ and $\nabla_{y}$the convolution kernels are given by:

$$\nabla_{x}= \left[ {\begin{aligned} 1 \\ 1 \\ 1 \\ 0 \end{aligned}} {\begin{aligned} 0 \\ 0 \\ 0 \\ 0 \end{aligned}}\begin{aligned} 0 \\ 0 \\ 0 \\ 0 \end{aligned} \begin{aligned} -1 \\ -1 \\ -1 \\ 0 \end{aligned} \right]$$

$$\nabla_{y}= \left[ {\begin{aligned} -1 \\ 0 \\ 0 \\ 1 \end{aligned}} {\begin{aligned} -1 \\ 0 \\ 0 \\ 1 \end{aligned}}\begin{aligned} -1 \\ 0 \\ 0 \\ 1 \end{aligned} \begin{aligned} 0 \\ 0 \\ 0 \\ 0 \end{aligned} \right]$$

In order to track PS, a tracking algorithm was implemented as previously described(Dharmaprani et al., 2019). New PS were defined as the detection of a PS not falling within the surrounding radius of $r$ of an existing PS for a duration of 𝜏. As default, 𝜏 was set to 10ms (20 frames) and $r$ set to 80 in computer simulated AF, 4 in basket AF, 2 in HD-grid AF and 20 in optically mapped AF to account for the varying spatial resolution of the mapped field.

To determine PS lifetime and inter-formation times, a look-up table indexing onset time, offset time and electrode location for each PS created(Dharmaprani et al., 2019). If the PS does not fall within a radius $r$ of another, the PS is given a new ID by incrementing the list of current PS IDs by 1. The look-up table will then record the current time stamp as the first time the PS is detected, and records all consequent time stamps it remains present. The look-up table also records the x and y locations of the PS on the phase map. For PS, this is given by a paired (x,y) coordinate value Using this look-up table information, the total time the is present (lifetime) can be measured, as well as the time taken between the creation of the current PS until the creation of the next consecutive PS (inter-formation time).

# S6 - AF Baseline Characteristics

6.a - Basket Catheter Cohort

The baseline characteristics of the recorded basket catheter cases are presented here. These cases have been used in previously published studies.(Dharmaprani et al., 2019)

| Age (years) | 61.1 $\pm$ 8.8 |
| --- | --- |
| Male, (%) | 66% |
| BMI (kg/m2) | 30.0 $\pm$ 5.2 |
| CHA2DS2VaSC | 2 $\pm$ 0.8 |
| Persistent AF, (%) | 62.5% |
| Paroxysmal AF, (%) | 37.5% |
| LVEF (%) | 58.1 $\pm$ 6.5 |
| LA Diameter | 29 $\pm$ 8.4 |

6.b - HD Grid Cohort

The baseline characteristics of the recorded HD grid cases used in this study are presented here.

| Age (years) | 57.2 $\pm$12.0 |
| --- | --- |
| Male, (%) | 50% |
| BMI (kg/m2) | 30.4 $\pm$3.5 |
| CHA2DS2VaSC | 1.6 $\pm$1.7 |
| Persistent AF, (%) | 30% |
| Paroxysmal AF, (%) | 70% |
| LVEF (%) | 60 $\pm$ 6.1 |
| LA Volume (ml/BSA) | 87.3 $\pm$ 12.2 |

**S7 - VF Cohort Baseline Characteristics**

The VF cohort clinical characteristics have been described in previous studies. (Nash et al., 2006a; Dharmaprani et al., 2022)

| **Patient** | **Age/Sex** | **CAD/AVD/MVD** | **LAD** | **Cx** | **RCA** | **EF, %** | **MI** | **Β-blockade** | **ACE** | **Others** |
| --- | --- | --- | --- | --- | --- | --- | --- | --- | --- | --- |
| **H055** | 60/M | AVD | N | N | N | 56 | No | No | No | Amlodipine |
| **H058** | 67/M | AVD | N | N | N | 67 | No | No | No | No |
| **H060** | 78/F | AVD | N | N | N | 64 | No | No | Yes | No |
| **H062** | 30/M | AVD | N | N | N | 66 | No | No | No | No |
| **H063** | 77/M | CAD | Mild | Mod | Mod |  | No | No | Yes | No |
| **H064** | 65/M | CAD | Mod | Mod | Mod | 52 | No | Yes | No | No |
| **H065** | 73/M | CAD | Mod | N | Mod | 41 | No | Yes | No | Amlopidine, Nicoandil |
| **H066** | 46/F | MVD | N | N | N | 80 | No | No | No | No |

**S8 – Rat Optical Mapping**

8.a - MI Surgery for RAT AF

This dataset has been described in previous publications.(Dharmaprani et al., 2019) Male Wistar rats weighing 200-275g were injected subcutaneously (SC) with preoperative buprenorphine (0.03 mg/kg) and anaesthetized with 2% isoflurane. Under endotracheal intubation and assisted ventilation, a left thoracotomy was performed, followed by ligation of the left anterior descending coronary artery with 6-0 silk. The thorax was sutured using a 3-0 silk and the skin was stapled using metal clips. Buprenorphine (0.03 mg/kg) was injected SC 6 and 12 hours postoperatively. Echocardiography was performed at baseline 1 day before the surgery, then 2 weeks later, to assess successful MI, and followed by final echocardiography 3 weeks following surgery. The same day, rats underwent transesophageal electrophysiological study (EPS).

8.b Rat VF

This dataset has been described in previous publications.(Ilkan et al., 2018; Dharmaprani et al., 2019; Strauss et al., 2019) In VF, rat hearts were retrogradely perfused with oxygenated Tyrodes solution (37^o^C, perfusion pressure 60mmHg). Recording duration was 2 seconds for n = 10 cases.

References

Aguilar, M., Feng, J., Vigmond, E., Comtois, P., and Nattel, S. (2017). Rate-dependent role of IKur in human atrial repolarization and atrial fibrillation maintenance. *Biophysical journal* 112(9)**,** 1997-2010.

Aliev, R.R., and Panfilov, A.V. (1996). A simple two-variable model of cardiac excitation. *Chaos, Solitons & Fractals* 7(3)**,** 293-301.

Bray, M.-A., and Wikswo, J.P. (2002a). Use of topological charge to determine filament location and dynamics in a numerical model of scroll wave activity. *IEEE transactions on biomedical engineering* 49(10)**,** 1086-1093.

Bray, M.A., and Wikswo, J.P. (2002b). Considerations in phase plane analysis for nonstationary reentrant cardiac behavior. *Physical review. E, Statistical, nonlinear, and soft matter physics* 65(5 Pt 1)**,** 051902.

Courtemanche, M., Ramirez, R.J., and Nattel, S. (1998). Ionic mechanisms underlying human atrial action potential properties: insights from a mathematical model. *American Journal of Physiology-Heart and Circulatory Physiology* 275(1)**,** H301-H321.

Dharmaprani, D., Jenkins, E.V., Quah, J.X., Lahiri, A., Tiver, K., Mitchell, L., et al. (2022). A governing equation for rotor and wavelet number in human clinical ventricular fibrillation: Implications for sudden cardiac death. *Heart Rhythm* 19(2)**,** 295-305.

Dharmaprani, D., Schopp, M., Kuklik, P., Chapman, D., Lahiri, A., Dykes, L., et al. (2019). Renewal Theory as a Universal Quantitative Framework to Characterize Phase Singularity Regeneration in Mammalian Cardiac Fibrillation. *Circulation: Arrhythmia and Electrophysiology* 12(12)**,** e007569.

Ilkan, Z., Strauss, B., Campana, C., and Akar, F.G. (2018). "Optical Action potential mapping in acute models of ischemia–reperfusion injury: probing the arrhythmogenic role of the mitochondrial translocator protein," in *Experimental Models of Cardiovascular Diseases*. Springer), 133-143.

Kneller, J., Zou, R., Vigmond, E.J., Wang, Z., Leon, L.J., and Nattel, S. (2002). Cholinergic atrial fibrillation in a computer model of a two-dimensional sheet of canine atrial cells with realistic ionic properties. *Circulation research* 90(9)**,** e73-e87.

Kuklik, P., Zeemering, S., Maesen, B., Maessen, J., Crijns, H.J., Verheule, S., et al. (2015). Reconstruction of instantaneous phase of unipolar atrial contact electrogram using a concept of sinusoidal recomposition and Hilbert transform. *IEEE Trans Biomed Eng* 62(1)**,** 296-302. doi: 10.1109/TBME.2014.2350029.

Nash, M.P., Mourad, A., Clayton, R.H., Sutton, P.M., Bradley, C.P., Hayward, M., et al. (2006a). Evidence for multiple mechanisms in human ventricular fibrillation. *Circulation* 114(6)**,** 536-542.

Nash, M.P., Mourad, A., Clayton, R.H., Sutton, P.M., Bradley, C.P., Hayward, M., et al. (2006b). Evidence for multiple mechanisms in human ventricular fibrillation. *Circulation* 114(6)**,** 536-542. doi: 10.1161/CIRCULATIONAHA.105.602870.

Pan, J., and Tompkins, W.J. (1985). A real-time QRS detection algorithm. *IEEE Trans. Biomed. Eng* 32(3)**,** 230-236.

Ravinutala, S. (2018). *Another flavour of the waiting time (or inspection) paradox* [Online]. Github. Available: <https://sidravi1.github.io/blog/2018/11/14/another-flavour-of-the-waiting-time-or-inspection-paradox> [Accessed 6th July 2021].

Roney, C.H., Cantwell, C.D., Qureshi, N.A., Chowdhury, R.A., Dupont, E., Lim, P.B., et al. (2017a). Rotor Tracking Using Phase of Electrograms Recorded During Atrial Fibrillation. *Ann Biomed Eng* 45(4)**,** 910-923. doi: 10.1007/s10439-016-1766-4.

Roney, C.H., Cantwell, C.D., Qureshi, N.A., Chowdhury, R.A., Dupont, E., Lim, P.B., et al. (2017b). Rotor tracking using phase of electrograms recorded during atrial fibrillation. *Annals of biomedical engineering* 45(4)**,** 910-923.

Shkurovich, S., Sahakian, A.V., and Swiryn, S. (1998). Detection of atrial activity from high-voltage leads of implantable ventricular defibrillators using a cancellation technique. *IEEE Transactions on Biomedical Engineering* 45(2)**,** 229-234.

Strauss, B., Sassi, Y., Bueno-Beti, C., Ilkan, Z., Raad, N., Cacheux, M., et al. (2019). Intra-tracheal gene delivery of aerosolized SERCA2a to the lung suppresses ventricular arrhythmias in a model of pulmonary arterial hypertension. *Journal of molecular and cellular cardiology* 127**,** 20-30.

Ten Tusscher, K., and Panfilov, A. (2003). Influence of nonexcitable cells on spiral breakup in two-dimensional and three-dimensional excitable media. *Physical Review E* 68(6)**,** 062902.

Vidmar, D., and Rappel, W.J. (2018). To the Editor- On the deformation and interpolation of phase maps. *Heart Rhythm* 15(2)**,** e3. doi: 10.1016/j.hrthm.2017.11.004.
